# High cytotoxicity of betulin towards fish and murine fibroblasts: Is betulin safe for nonneoplastic cells?

**DOI:** 10.1186/s12917-021-02905-x

**Published:** 2021-05-25

**Authors:** Joanna Małaczewska, Edyta Kaczorek-Łukowska, Barbara Kazuń

**Affiliations:** 1 Department of Microbiology and Clinical Immunology, Faculty of Veterinary Medicine, University of Warmia and Mazury in Olsztyn, Oczapowskiego Street 13, 10-719 Olsztyn, Poland; 2 Department of Fish Pathology and Immunology, Stanisław Sakowicz Inland Fisheries Institute, Olsztyn, Poland

**Keywords:** betulin, cytotoxicity, fibroblasts, NIH/3T3, BF-2

## Abstract

**Background:**

Betulin, a natural pentacyclic triterpene with the lupane structure that is present in significant amounts in the outer bark of birch, is known for its broad array of biological and pharmacological properties. Betulin has attracted attention as a potential, natural-origin antimicrobial substance. The literature describes it as selectively toxic to neoplastic cells but safe for normal cells.

The research aim was to evaluate the basal cytotoxicity of betulin towards fish (BF-2) and murine (NIH/3T3) fibroblasts. We used four colorimetric tests that provide a preliminary evaluation of possible mechanisms of the cytotoxicity of a compound to assess the degree of the toxicity of betulin after 24, 48 and 72 h of incubation with cells: the MTT assay (mitochondrial activity assessment), the NRU assay (lysosomal membrane integrity assessment), the LDH assay (cellular membrane integrity assessment) and the SRB assay (total cellular protein content determination).

**Results:**

The results revealed an exceptionally high sensitivity of mitochondria to the effect of betulin, with the other endpoints being less sensitive. Although murine fibroblasts were more vulnerable to the toxic effect of betulin than fish fibroblasts, the betulin CC_50_ values for both cell lines were comparable with analogous IC_50_ values determined by other researchers in studies involving cancerous cells.

**Conclusions:**

The results indicate the need to verify the claim about the selective toxicity of betulin towards malignant cells and to conduct safety/toxicity tests before any potential therapeutic use of betulin in veterinary medicine.

**Supplementary Information:**

The online version contains supplementary material available at 10.1186/s12917-021-02905-x.

## Background

Betulin (betulinol, betuline, betulinic alcohol, BE) is a plant-derived, natural pentacyclic triterpene with the lupane structure. It has been extracted from many plant species, but it is most abundant in the outer bark of birch, which also contains the natural BE derivative betulinic acid (BA), a product of betulin oxidation [1–4]. Numerous scientific studies have confirmed the medicinal properties of birch and other members of the Betulaceae family, which have been known in traditional medicine for ages. To date, a broad spectrum of biological and pharmacological characteristics of BE and BA have been described, including anticancer, antiviral, antibacterial, antifungal, antimalarial, antiallergic, antiangiogenic, anti-inflammatory, antifibrotic, anticonvulsant, hepatoprotective and chemopreventive properties [1–4]. In the age of increasing antimicrobial resistance and emergence of new multidrug-resistant pathogens there is a constant search for new and safe antimicrobials with novel modes of action and reduced risk for the development of resistance amongst pathogens [5, 6]. Oloyede et al. [7] have recently proved that antibacterial activity of betulin towards *Escherichia coli*, *Pseudomonas aeruginosa* and *Staphylococcus aureus* is the result of oxidative stress caused by this compound in bacterial cells. Despite its promising pharmacological characteristics, betulin is not being used in veterinary practice so far. Reports on the antibacterial, antifungal and antiviral properties of betulin [7–10] encouraged us to undertake a study on the antimicrobial activity of this natural compound towards some veterinary pathogens, both bacterial and viral pathogens. To our disappointment, our research did not confirm the antimicrobial activity of BE towards any of the tested pathogens (data not shown). However, we were surprised by the high level of toxicity of betulin towards cell lines that were used to evaluate the antiviral activity of BE. Despite published reports on the toxicity of BE towards normal cells [11–14], a lingering belief in the literature is that betulin, similar to betulinic acid [15], is selectively toxic towards neoplastic cells but is only weakly toxic towards normal cells [1–3]. To date, the effect of betulin on normal cells (mainly fibroblasts) has rarely been tested and typically only using a single cytotoxicity assay; these cells are usually treated as a reference for neoplastic cell lines used for the *in vitro* assessment of the anticancer activity of betulin [11–14, 16, 17]. Established murine and human fibroblast cell lines are amongst the most frequently used cells in basal cytotoxicity testing [18, 19]. However, at least two different cytotoxicity assays should be performed to monitor different endpoints of basal cytotoxicity of a compound [19]. The Interagency Coordinating Committee on the Validation of Alternative Methods (ICCVAM) recommends using well established basal cytotoxicity assays that have good interlaboratory reproducibility and determine either cell proliferation or cell viability (e.g. NRU, MTT or XTT assays) [18]. To the best of our knowledge, none of the references in the available literature describe the effect of betulin on fish cells. Fish cell lines are not recommended by ICCVAM for basal cytotoxicity testing due to relatively long doubling time [18]. Castaño and Gómez-Lechón [20] proved, however, that there is good linear correlation of IC_50_ values between fish and mammalian cells. Fish cells are better predictors for chemicals toxicity towards aquatic organisms than mammalian cells. Fish BF-2 fibroblasts were proven to be a sensitive indicator for evaluating aquatic ecotoxicology [21]. They were used successfully for basal cytotoxicity testing of graphene oxide and nickel nanoparticles [21, 22].

A serious limitation to testing the biological activity of betulin is its poor solubility in aqueous media resulting from its lipophilic nature [1–4]. Dimethyl sulfoxide (DMSO) is predominantly used as a solvent in *in vitro* studies on betulin. DMSO is an organic polar aprotic solvent that is frequently used to dissolve polar and nonpolar hydrophobic compounds. It is preferred due to its miscibility in water and some other organic solvents. However, some studies indicate a relatively high level of toxicity of DMSO towards different cell types, including fibroblasts [23, 24]. Vehicles used in cell biology should not be toxic to cells; thus, cytotoxicity studies of organic solvents are necessary, and the most validated and standardized test to evaluate the safety of using a solvent is the MTT assay [25].

Considering this information, after determining the biocompatibility of DMSO with the MTT assay, we decided to test the basal cytotoxicity of betulin towards two animal fibroblast cell lines, NIH/3T3 (murine fibroblasts) and BF-2 (fish fibroblasts), using four different colorimetric cytotoxicity assays that analysed the effect of a tested substance on the activity of mitochondria (MTT assay), cellular (LDH assay) and lysosomal (NRU assay) membrane integrity, and the total cellular protein content (SRB assay).

## Results

### Betulin solubility and vehicle (DMSO) cytotoxicity

In our experiment, the maximum solubility of betulin in DMSO at room temperature was 2500 µg/mL. We were able to dissolve 5000 µg of betulin in 1 mL of DMSO, although it required heating, and the solution remained unstable. After mixing with the cell culture medium, the solution immediately became slightly turbid. Therefore, a 2500 µg/mL stock solution of betulin in DMSO and a series of 2-fold increasing dilutions of the stock solution in cell culture media were prepared (final working concentrations of 0.244, 0.488, 0.976, 1.95, 3.9, 7.8, 15.625 and 31.25 µg/mL).

**Fig. 1:**
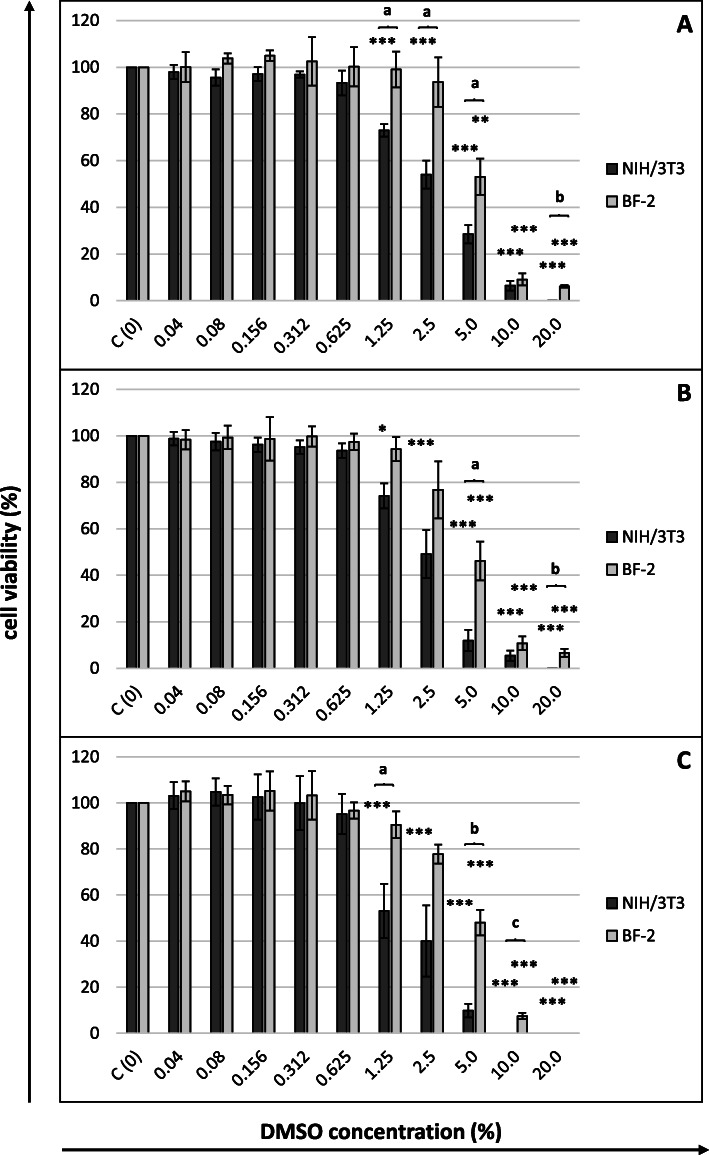
Cytotoxicity of DMSO after (**A**) 24, (**B**) 48 and (**C**) 72 h of cells exposure (MTT assay) Cell viability expressed as the percentage of control (untreated) cell viability. DMSO concentrations in %. All data expressed as means ± SD (standard deviation) for *n* = 3 independent experiments. Asterisks refer to statistically significant differences between control and treatments analyzed by one-way ANOVA followed by Dunnett’s posttest; ** *p*<0.01, *** *p*<0.001. Lower case letters indicate significant differences between the two cell lines at the same time points determined using Student’s *t*-test; ^a^*p*<0.05, ^b^*p*<0.01, ^c^*p*<0.001

The cytotoxic effect of DMSO towards both cell lines used in our experiment was dose-dependent, and time-dependent only in relation to murine fibroblasts. Murine fibroblasts proved to be more sensitive (CC_50_ values ranging between 1.635 and 2.989 %) than fish fibroblasts (CC_50_ values ranging between 4.434 and 5.031 %) (Fig. 1; Table 1).

**Table 1:** CC_50_ values for vehicle (DMSO) and betulin

cell line	DMSO (MTT)			betulin								
				**MTT**			**NRU**			**SRB**		
	24 h	48 h	72 h	24 h	48 h	72 h	24 h	48 h	72 h	24 h	48 h	72 h
**NIH/3T3**	2.989 ± 0.159	2.374 ± 0.395	1.635 ± 0.423	1.532 ± 0.005	1.585 ± 0.64	1.853 ± 0.647	˃15.625	7.329 ± 2.942	12.954 ± 2.942	10.87 ± 7.338	4.051 ± 1.242	3.756 ± 0.572
**BF-2**	5.031 ± 0.423	4.434 ± 0.717	4.794 ± 0.337	3.394 ± 0.903	2.751 ± 1.051	3.021 ± 1.034	27.615 ± 9.006	16.462 ± 3.599	14.516 ± 3.41	>31.25	>31.25	>31.25

DMSO concentrations in %, betulin concentrations in µg/mL. CC_50_ (50 % cytotoxic concentration) values expressed as means ± SD (standard deviation) for *n* = 3 independent experiments. CC_50_ values were calculated using the Quest Graph™ ED50 Calculator (AAT Bioquest, Inc.)

The maximum tolerable DMSO concentration was deemed to be the concentration at which the viability of cells exceeded 90 % throughout the experiment, and this concentration was 0.625 % DMSO for NIH/3T3 cells and 1.25 % DMSO for BF-2 cells. These concentrations of the vehicle corresponded to betulin concentrations of 15.625 and 31.25 µg/mL, respectively. Hence, the range of tested betulin concentrations in our subsequent experiments was slightly different for the two cell lines and equalled 0.244–15.625 µg/mL (7 concentrations) for murine fibroblasts and 0.244–31.25 µg/mL (8 concentrations) for fish fibroblasts.

### Betulin cytotoxicity

The cytotoxicity of betulin towards NIH/3T3 (concentrations 0.244–15.625 µg/mL) and BF-2 cells (concentrations 0.244–31.25 µg/mL) was evaluated using four different colorimetric tests after 24, 48 and 72 h of incubation, and the CC_50_ values obtained from the MTT, NRU and SRB assays are shown in Table 1. In all three assays, murine fibroblasts were characterized by a higher sensitivity to the toxic effect of betulin than fish fibroblasts (lower CC_50_ values). In most of the experiments, the level of betulin cytotoxicity increased up to 48 h, after which it became stabilized (similar CC_50_ values were observed after 48 and 72 h). The only exception was the results of the MTT assay applied to NIH/3T3 cells. The lowest CC_50_ values for both cell lines were obtained in the MTT assay, regardless of the incubation time, which implies the highest sensitivity of this cytotoxicity assay for betulin. The CC_50_ values determined in the NRU assay were several times higher than those obtained in the MTT assay. In the case of NIH/3T3 cells, the results obtained in the SRB assay corresponded better to the results of the MTT assay than to those obtained with the NRU test. The results were different when the assays were applied to BF-2 cells. The viability of fish cells in this assay upon exposure to the tested range of betulin concentrations did not decrease below 50 %, which made it impossible to determine the CC_50_ values.

**Fig. 2:**
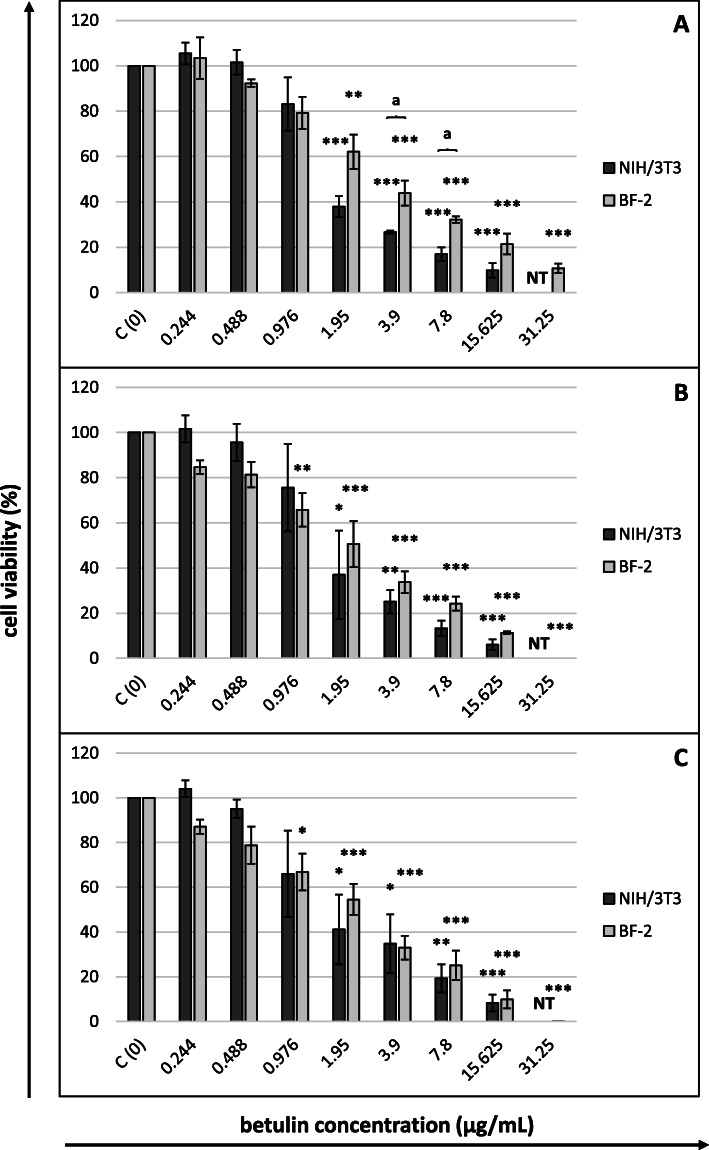
Cytotoxicity of betulin after (**A**) 24, (**B**) 48 and (**C**) 72 h of cells exposure (MTT assay) Cell viability expressed as the percentage of control (untreated) cell viability. Betulin concentrations in µg/mL. All data expressed as means ± SD (standard deviation) for *n* = 3 independent experiments. Asterisks refer to statistically significant differences between control and treatments analyzed by one-way ANOVA followed by Dunnett’s posttest; * *p*<0.05, ** *p*<0.01, *** *p*<0.001. Lower case letters indicate significant differences between the two cell lines at the same time points determined using Student’s *t*-test; ^a^*p*<0.05. NT – betulin concentration not tested (for NIH/3T3 cells)

**Fig. 3:**
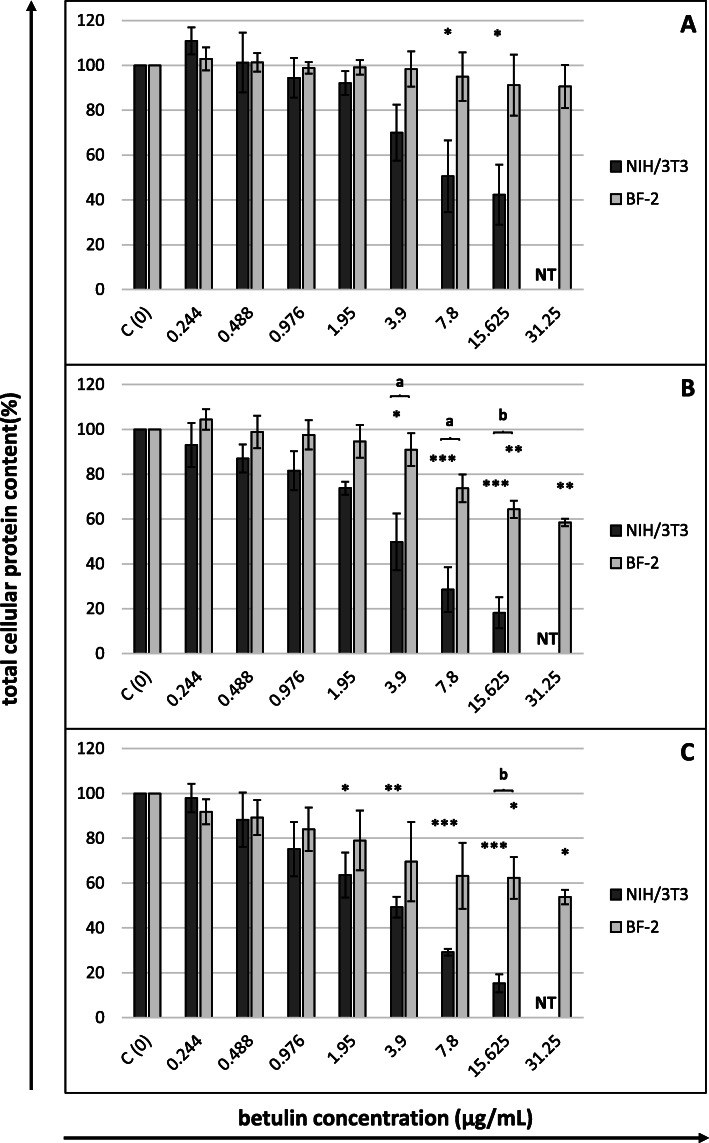
Cytotoxicity of betulin after (**A**) 24, (**B**) 48 and (**C**) 72 h of cells exposure (SRB assay) Cytotoxicity expressed as the percentage of the total cellular protein content in control (nontreated) cells. Betulin concentrations in µg/mL. All data expressed as means ± SD (standard deviation) for *n* = 3 independent experiments. Asterisks refer to statistically significant differences between control and treatments analyzed by one-way ANOVA followed by Dunnett’s posttest; * *p*<0.05, ** *p*<0.01, *** *p*<0.001. Lower case letters indicate significant differences between the two cell lines at the same time points determined using Student’s *t*-test; ^a^*p*<0.05, ^b^*p*<0.01. NT – betulin concentration not tested (for NIH/3T3 cells)

**Fig. 4:**
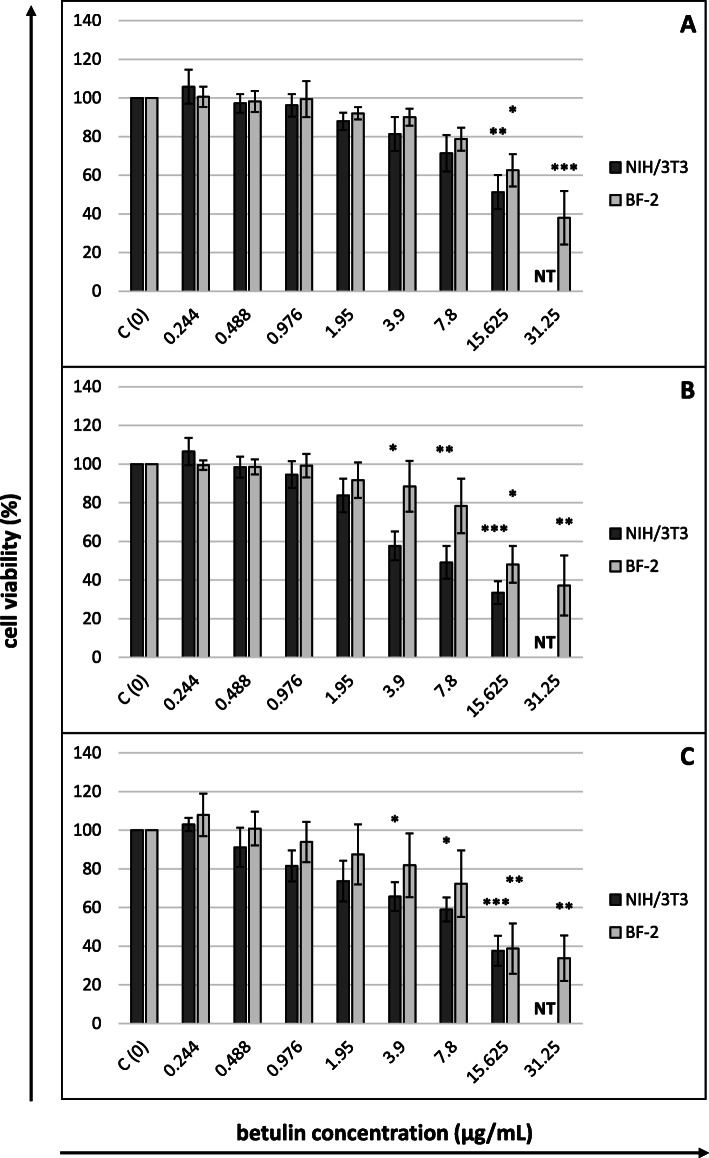
Cytotoxicity of betulin after (**A**) 24, (**B**) 48 and (**C**) 72 h of cells exposure (NRU assay) Cell viability expressed as the percentage of control (untreated) cell viability. Betulin concentrations in µg/mL. All data expressed as means ± SD (standard deviation) for *n* = 3 independent experiments. Asterisks refer to statistically significant differences between control and treatments analyzed by one-way ANOVA followed by Dunnett’s posttest; * *p*<0.05, ** *p*<0.01, *** *p*<0.001. NT – betulin concentration not tested (for NIH/3T3 cells)

**Fig. 5:**
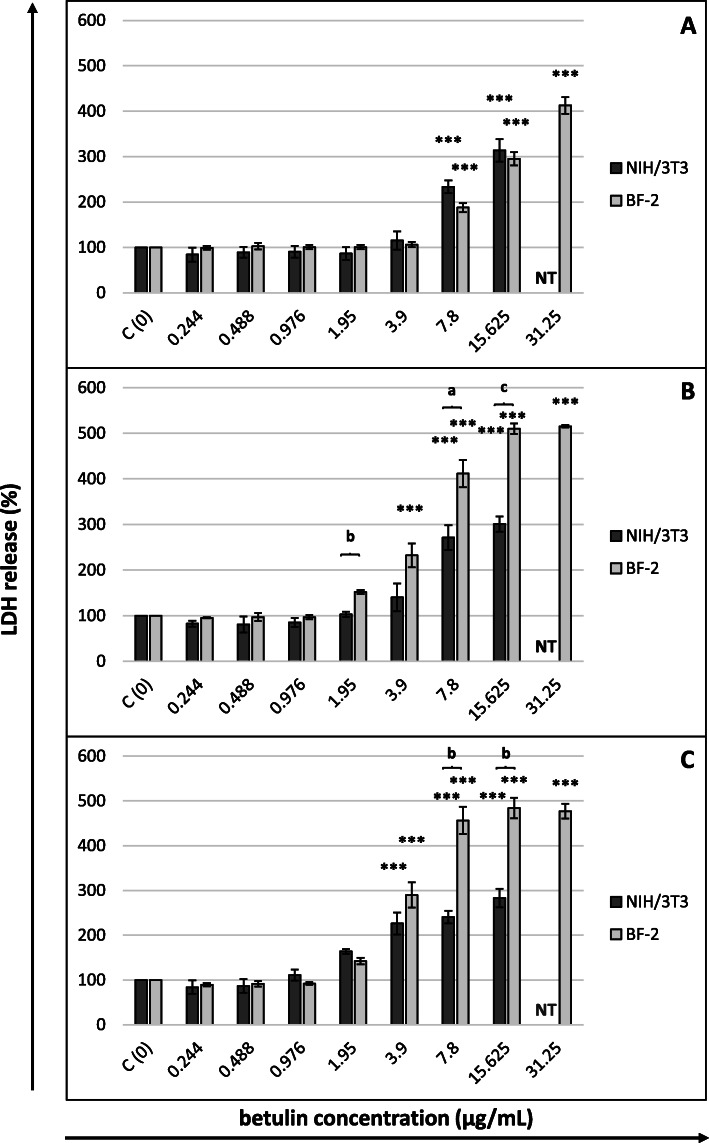
Cytotoxicity of betulin after (**A**) 24, (**B**) 48 and (**C**) 72 h of cells exposure (LDH assay) Cytotoxicity expressed as the percentage of LDH leakage to the culture media in control (nontreated) cells. Betulin concentrations in µg/mL. All data expressed as means ± SD (standard deviation) for *n* = 3 independent experiments. Asterisks refer to statistically significant differences between control and treatments analyzed by one-way ANOVA followed by Dunnett’s posttest; *** *p*<0.001. Lower case letters indicate significant differences between the two cell lines at the same time points determined using Student’s *t*-test; ^a^*p*<0.05, ^b^*p*<0.01, ^c^*p*<0.001. NT – betulin concentration not tested (for NIH/3T3 cells)

Figures 2, 3, 4 and 5 present the dose-response curves for the four assays after 24, 48 and 72 h of exposure of NIH/3T3 and BF-2 cells to betulin. Similar levels of sensitivity were achieved for both cell lines only in the MTT assay (Fig. 2). A statistically significant decrease in the viability of fibroblasts after 24 h of incubation was noted at betulin concentrations ≥ 1.95 µg/mL. This threshold value persisted until the termination of the experiment using mouse cells. With respect to fish cells, however, starting at 48 h of incubation, a considerable decrease in the viability of these cells was also observed with a lower betulin concentration (0.976 µg/mL), although the CC_50_ values for BF-2 lines were approximately twice as high as those for murine fibroblasts. The underlying cause of these differences was higher values for the standard deviations of the results obtained from NIH/3T3 cells. The results of the MTT assay corresponded well with the microscopic observations of the cells. Regarding the cells incubated with betulin concentrations that considerably decreased their viability in the MTT assay, morphological changes such as cell shrinkage, rounding and loss of adhesion (at higher concentrations) were observed under an inverted phase contrast microscope (Additional files 1 and 2).

The results of the MTT assay using NIH/3T3 cells best corresponded to the SRB assay results (Fig. 3), where after a 72 h incubation, a considerable decrease in the total cellular protein content was observed in cells exposed to betulin concentrations ≥ 1.95 µg/mL. Simultaneously, the lowest sensitivity of this assay was observed in BF-2 cells, and a considerable decrease in the number of these cells was caused by betulin concentrations of at least 15.625 µg/mL.

Likewise, the sensitivity of the NRU assay was higher for murine cells than for fish cells (Fig. 4). A considerable decrease in the viability of NIH/3T3 cells was observed beginning at 48 h of incubation with betulin concentrations ≥ 3.9 µg/mL, whereas in the case of BF-2 cells, these effects occurred at concentrations of at least 15.625 µg/mL, regardless of the time of incubation.

For the LDH assay, a considerable leakage of LDH from both types of cells was observed at betulin concentrations ≥ 3.9 µg/mL (after a 72 h incubation in the case of murine cells and after 48 h in the case of fish cells), although the readings from this test were higher when applied to fish fibroblasts, which implies a higher sensitivity of the cytoplasmic membrane of these cells (Fig. 5).

## Discussion

As mentioned above, due to the poor solubility of betulin, DMSO was used as a solvent and we determined the level of its toxicity towards the cell lines used in the experiment and subsequently selected a range of concentrations of betulin that we would be able to test under the experimental conditions. Fish cells were characterized by lower sensitivity to the toxic effect of DMSO than murine fibroblasts (maximum tolerable concentrations of 1.25 and 0.625 %; CC_50_ values after 72 h of 4.794 and 1.635 %, respectively). The results we obtained correlate with published data to some extent. Adler et al. [23], who tested the toxicity of DMSO towards murine Balb/3T3 fibroblasts using the MTT assay, determined an IC_50_ (50 % inhibitory concentration) value of 2.75 %. In turn, Singh et al. [24] used the trypan blue dye exclusion assay and found that DMSO concentrations ranging from 0.5 to 3 % led to a reduction in the viability of GSF3.2 goat skin fibroblasts in a dose-dependent manner. DMSO concentrations ranging from 5 to 9 % killed all GSF3.2 cells, and at concentrations less than 0.1 %, the cited authors observed a significant increase in the number of live cells. In our study, the absence of any measurable activity of mitochondria was observed in murine and fish fibroblasts at concentrations as high as 10 and 20 % DMSO, respectively. We also did not observe a higher viability of cells incubated with low concentrations of the solvent.

In our experiment, murine fibroblasts were more sensitive than fish cells to the toxic effect of betulin, as indicated by the values of betulin CC_50_ concentrations for both cell lines determined using the MTT, SRB and NRU assays. The same relationship related to the sensitivity of the cells to the harmful influence of the vehicle (DMSO). The finding that different cell lines manifest different levels of sensitivity to both betulin and DMSO is consistent with published data [2, 3, 24].

The considerable differences in CC_50_ concentrations observed depending on the applied assay are also unsurprising because these assays evaluate different parameters (see the Methods section). Because the MTT assay detects only viable cells while the SRB assay does not distinguish between viable and dead cells, the CC_50_ values of compounds tested using the SRB assay are usually higher [26]. The results from the present study clearly indicate the particularly high sensitivity of mitochondria to the effect of betulin, with other endpoints being less sensitive. Similar observations were reported by Pfarr et al. [27], who analysed the cytotoxicity of betulin (concentrations of 0-150 µM) towards two melanoma cell lines (B164A5 and B16F10). B16 cells treated with 150 µM betulin exhibited a trypan blue exclusion capacity of 60 %, while an almost complete lack of cell mitochondrial activity in the MTT assay was observed beginning at a low betulin concentration of 15 µM. According to the authors, the results implicated an arrest in proliferation without killing the cells. A similar effect on normal cells was reported by Lin et al. [28]. At concentrations up to 5.12 µg/mL, betulin did not affect the integrity of the cytoplasmic membrane of porcine chondrocytes in an LDH assay, while a considerable decrease in the viability of cells was observed in the MTT assay at a concentration of 0.02 µg/ml. For neoplastic cells, the main mechanism responsible for the cytotoxic activity of betulin is presumed to be the stimulation of the intrinsic pathway (mitochondrion-dependent) of cell apoptosis [3]. An analogous mechanism may occur in noncancerous cells.

The comparison of our results with those reported in the literature is challenging because, as we have already mentioned, the cytotoxicity of betulin towards normal cells has not been the subject of many studies conducted to date, and few studies have simultaneously applied various cytotoxicity assays. Two articles authored by the same team of researchers [13, 14] have examined the same line of murine fibroblasts as used in our research (NIH/3T3). However, these scholars did not determine the CC_50_ concentration; instead, they measured the inhibitory activity of betulin by incubating cells with a concentration of 20 µM (8.9 µg/mL) for 72 h, after which they determined the viability of these cells using the MTT assay. The inhibitory effect of betulin on murine fibroblasts in both of these reports was approximately 30 %. In our experiment, the most approximate concentration is listed above, as after 72 h of incubation, a concentration of 7.8 µg/mL caused an approximately 80 % decrease in cell viability in the MTT assay. The aforementioned authors did not test betulin from the birch tree but derived it from two other plants (*Belamcanda chinensis* and *Cyrtomium fortumei*, respectively), which may explain the observed discrepancies in the sensitivity of cells. In a study by Boryczka et al. [16], murine fibroblasts (Balb 3T3 line) were also distinguished by a lower sensitivity to the toxic effect of betulin. The IC_50_ value measured in an SRB assay equalled 47.3 µg/ml. Although the cited researchers did not provide specific data on the duration of the incubation of cells with betulin, the CC_50_ of betulin obtained in our study using the SRB assay ranged from 3.756 to 10.87 µg/ml, depending on how long the cells were incubated with the compound. The only difference between the NIH/3T3 and Balb 3T3 lines is that cells originate from different strains of mice, and this difference was very unlikely to be the cause of such considerable discrepancies in the betulin CC_50_ values. On the other hand, substantial differences in the IC_50_ values of betulin determined in exactly the same cancer cell lines were observed in research conducted by different authors [3].

Other studies on the toxicity of betulin towards fibroblasts were conducted using human cells. All possible differences in the results achieved in this study compared to the present study obviously may arise from the fact that these are cells from different species. Nonetheless, we were still surprised to note that the actual differences were not large. The IC_50_ of betulin in human lung fibroblasts (WI 38) determined using the trypan blue exclusion test after a 72 h incubation was 15.2 µM (ca. 6.75 µg/ml) [11], similar to the values we obtained in some of our experiments. A result that is even closer to ours was reported by Gauthier et al. [12] from their study on human skin fibroblasts (WS1) after a 48 h incubation of cells with betulin. The IC_50_ determined in this study using the resazurin reduction test (RRT) was 3.58 µM (ca. 1.6 µg/ml). The RRT is an assay that evaluates the metabolic activity of mitochondria, which resembles the MTT assay employed in our study, and the results reported by the cited authors correspond perfectly with the outcome of the MTT assay performed in our study. Likewise, in a study conducted to assess the effect of betulin on human skin fibroblasts (HSF), Rzeski et al. [17] concluded that after 24-h exposure of cells, betulin concentrations up to 5 µM (ca. 2.2 µg/ml) were not toxic, but concentrations ≥ 10 µM (ca. 4.4 µg/ml) caused considerable LDH leakage into the culture medium. In our study, after 24 h of incubation, regardless of the cell line, substantial LDH leakage was induced by 7.8 µg/ml betulin, whereas the dose of 3.9 µg/ml was toxic towards both types of cells after 48 or 72 h of exposure.

The main objects of *in vitro* studies involving betulin conducted to date have been various types of neoplastic cells, as reviewed by Król et al. [3] and Hordyjewska et al. [2]. The authors of these comprehensive reviews highlight considerable differences in the anticancer effects of betulin, depending on the cancer cell type, with IC_50_ values ranging from 1.1 to more than 100 µg/ml. The betulin CC_50_ values determined in our study using murine and fish fibroblasts indicate the high sensitivity of normal cells, approaching the maximum threshold of sensitivity of neoplastic cells. Our observations confirm the results of studies described in several of the papers cited earlier in this article, where fibroblasts were treated as a point of reference to the tested neoplastic lines [11–14]. In their study, Hata et al. [11] reported that the IC_50_ values of betulin towards three leukaemia (HL60, U937, and K562), two melanoma (G361 and SK-MEL-28) and two neuroblastoma (GOTO and NB-1) cell lines were 14.7, 14.4, 14.5, 12.4, 16.2, 17.1 and 16.5 µM, respectively, whereas the IC_50_ for human lung fibroblasts (WI 38) equalled 15.2 µM. Furthermore, Gauthier et al. [12] reported that human skin fibroblasts (WS1) were characterized by a higher sensitivity to betulin than the tested neoplastic lines A-549, DLD-1 (human cancer) and B16-F1 (mouse melanoma). In this study, the IC_50_s of betulin were 3.58, 3.8, 6.6 and 13.8 µM, respectively. Two other papers provided similar data concerning the sensitivity of fibroblasts and neoplastic cells, although the IC_50_ was not determined in these experiments [13, 14]. The inhibitory activity of betulin at a concentration of 20 µM towards the neoplastic lines MGC-803 (stomach cancer), Bcap-37 (breast cancer), MCF-7 (breast cancer), PC3 (prostate cancer) and A375 (melanoma) reached 43.7–45.1, 53.2, 53.2, 17.3–18.4 and 33.9 %, respectively, while it reached approximately 29.8–33.5 % for the NIH/3T3 murine fibroblasts. Only a study by Boryczka et al. [16] reported a higher IC_50_ of betulin in the normal murine fibroblast Balb3T3 (47.3 µg/ml) than in the neoplastic lines SW707 (colorectal adenocarcinoma), CCRF/CEM (leukaemia), T47D (breast cancer), P388 (murine leukaemia), for which the IC_50_ values were 22.9, 10.9, 32.4 and 5.5 µg/ml, respectively. However, the authors cited above used two different cytotoxicity assays: the MTT assay for leukaemia cells and SRB assay for other cell types. Any comparison of the IC_50_ values obtained with the MTT and SRB assays that are so different from each other appears difficult, and this problem is distinctly manifested in the results of our present research.

## Conclusions

The research presented above revealed the high level of cytotoxicity of betulin towards animal fibroblasts, where cellular mitochondria were shown to be particularly vulnerable to this compound. The research results suggest that the claim about selective toxicity of betulin towards malignant cells, which still persists in the literature, must be verified. Thus, intensive safety/toxicity tests are necessary before considering any potential therapeutic use of betulin in veterinary medicine.

## Methods

### Chemicals and reagents

Betulin (≥ 98 % purity), Dulbecco’s modified Eagle’s medium (DMEM), minimum essential medium (Eagle, EBSS), foetal bovine serum, antibiotic-antimycotic solution, nonessential amino acids (NEAAs), glutamine, Triton X-100, 3-(4,5-dimethylthiazol-2-yl)-2,5-diphenyl tetrazolium bromide (MTT), neutral red based *in vitro* toxicology assay kit (TOX4), lactic dehydrogenase based *in vitro* toxicology assay kit (TOX7) and sulforhodamine B based *in vitro* toxicology assay kit (TOX6) were purchased from Sigma-Aldrich, Poland. Dimethyl sulfoxide (DMSO) was purchased from Chempur, Poland.

### Compound preparation

A stock solution of betulin was prepared in DMSO at a concentration of 2500 µg/mL, and working solutions were prepared in cell maintenance media (serum-free) at final concentrations of 0 (control cells), 0.244, 0.488, 0.976, 1.95, 3.9, 7.8, 15.625 and 31.25 µg/mL immediately before use in cell culture.

### Cell lines

NIH/3T3 (ATCC CRL-1658) mouse embryonic fibroblasts were grown in Dulbecco’s modified Eagle’s medium (DMEM) supplemented with 2 mM glutamine, 10 % foetal bovine serum and a 1 % antibiotic-antimycotic solution at 37 °C in a humidified atmosphere with 5 % CO_2_.

BF-2 (ATCC CCL-91) bluegill caudal trunk fibroblasts were grown in minimum essential medium (Eagle, EBSS) supplemented with 1 % nonessential amino acids (NEAAs), 10 % foetal bovine serum and 1 % antibiotic-antimycotic solution at 23 °C in a humidified atmosphere with 5 % CO_2_.

NIH/3T3 and BF-2 cells were seeded in 96-well plates at a density of 1 × 10^4^ or 2 × 10^4^ cells per well, respectively. After 24 h of incubation at an appropriate temperature, cells were exposed to betulin at the concentrations mentioned above for 24, 48 and 72 h. Control (untreated) cells served as the negative control and a reference point. Triton X-100 treated cells served as the positive internal control in MTT, NRU and LDH assays. Due to the specific character of SRB assay, there was no positive control in this test. After 24, 48 and 72 h of treatment, the four different cytotoxicity assays were performed. All experiments were repeated three times.

### Cytotoxicity assays

Four *in vitro* cytotoxicity colorimetric assays were conducted to identify possible mechanisms of betulin cytotoxicity, namely, the MTT reduction assay, the neutral red uptake assay (NRU), the lactate dehydrogenase leakage assay (LDH) and the sulforhodamine B assay (SRB).

#### MTT assay

The MTT assay is used to estimate mitochondrial damage, since it determines the activity of mitochondrial enzymes. These enzymes reduce the water-soluble tetrazolium dye MTT to insoluble purple formazan, which accumulates inside viable cells [29].

The MTT assay was conducted using the protocol described by Mosmann [29], with some modifications described in our previous article [30]. The results are reported as the percentage of control (nontreated) cell viability.

The MTT assay was used to determine the cytotoxicity of both betulin and the vehicle (DMSO).

#### NRU assay

The NRU assay evaluates lysosomal membrane integrity. It measures neutral red dye accumulation within lysosomes of viable cells. This phenomenon depends on the capacity of viable cells to maintain a pH gradient; hence, dead cells are unable to retain the dye [31].

The NRU assay was performed using a commercially available kit (TOX4) (Sigma-Aldrich, Poland) according to the manufacturer’s protocol. The full description of the test procedure was also provided in a published article [30]. The results obtained are reported as the percentage of control (nontreated) cell viability.

#### LDH assay

The LDH assay measures leakage of the stable cytosolic enzyme lactate dehydrogenase into the culture medium, which is an indicator of irreversible cell membrane damage and cell death [32].

The LDH assay was performed using a commercially available kit (TOX7) (Sigma-Aldrich, Poland) according to the manufacturer’s protocol. The full description of the test procedure was also reported previously [30]. The results obtained are reported as the percentage of LDH leakage to the culture media in control (nontreated) cells.

#### SRB assay

The sulforhodamine B colorimetric assay measures the cellular protein content, and the assay results are proportional to the number of cells. This assay has been used to determine the cell density; however, it does not distinguish between living and dead cells [33].

The SRB assay was performed using a commercially available kit (TOX6) (Sigma-Aldrich, Poland) according to the manufacturer’s protocol. In this assay, SRB binds to protein components of trichloroacetic acid-fixed cells, and the protein-bound dye is extracted with Tris. The results obtained are presented as the percentage of the total cellular protein content in control (nontreated) cells.

### Statistical analysis

All experiments were repeated three times. The results are presented as the mean values ± SD (standard deviation). Statistical analyses were performed using GraphPad Prism software (GraphPad Software, USA). Data were subjected to one-way analysis of variance (ANOVA). Dunnett’s posttest was used to determine differences between control and betulin-exposed cells at the same time points. Differences between the two cell lines at the same time points were determined using Student’s *t*-test. The 50 % cytotoxic concentrations (CC_50_, defined as the concentration of the compound that reduced cell viability by 50 %) were calculated using the Quest Graph™ ED50 Calculator (AAT Bioquest, Inc.) [34].

## Supplementary information

**Additional file 1.** Representative morphology of BF-2 cells treated for 72 h with different concentrations of betulin (A) control (untreated) (B) betulin 0.976 µg/mL (C) betulin 3.9 µg/mL (D) betulin 15.625 µg/mL (magnification, x100) (PDF).

**Additional file 2.** Representative morphology of NIH 3T3 cells treated for 72 h with different concentrations of betulin (A) control (untreated) (B) betulin 0.976 µg/mL (C) betulin 3.9 µg/mL (D) betulin 15.625 µg/mL (magnification, x100) (PDF).

## Data Availability

All data generated or analyzed during this study are available from the corresponding author on reasonable request.

## Abbreviations

BE: Betulin
BA: Betulinic acid
MTC: Maximum tolerable concentration
DMSO: Dimethyl sulfoxide
NIH/3T3: Mouse embryonic fibroblasts
BF-2: Bluegill caudal trunk fibroblasts
DMEM: Dulbecco’s modified Eagle’s medium
NEAAs: Nonessential amino acids
MTT: 3-(4,5-dimethylthiazol-2-yl)-2,5-diphenyl tetrazolium bromide
NRU: The neutral red uptake assay
LDH: The lactate dehydrogenase leakage assay
SRB: The sulforhodamine B assay
CC_50_: 50% cytotoxic concentration
IC_50:_: 50% inhibitory concentration

## References

[1] Csuk R. Targeting cancer by betulin and betulinic acid. In: Chen G, Lai P (eds) Novel Apoptotic Regulators in Carcinogenesis. Springer, Dordrecht. 2012; 10.1007/978-94-007-4917-7_11

[2] Hordyjewska A, Ostapiuk A, Horecka A, Kurzepa J (2019). Betulin and betulinic acid: triterpenoid derivatives with a powerful biological potential. Phytochem Rev.

[3] Król SK, Kiełbus M, Rivero-Müller A, Stepulak A. Comprehensive review on betulin as a potent anticancer agent. Biomed Res Int. 2015;584189. 10.1155/2015/584189 PMC4383233 25866796

[4] Sami A, Taru M, Salme K, Jari YK (2006). Pharmacological properties of the ubiquitous natural product betulin. Eur J Pharm Sci.

[5] Aslam B, Wang W, Arshad MI, Khurshid M, Muzammil S, Rasool MH, Nisar MA, Alvi RF, Aslam MA, Qamar MU, Salamat MKF, Baloch Z (2018). Antibiotic resistance: a rundown of a global crisis. Infect Drug Resist.

[6] Pathania R, Brown ED (2008). Small and lethal: searching for new antibacterial compounds with novel modes of action. Biochem Cell Biol.

[7] Oloyede HO, Ajiboye HO, Salawu MO, Ajiboye TO (2017). Influence of oxidative stress on the antibacterial activity of betulin, betulinic acid and ursolic acid. Microb Pathog.

[8] Pavlova NI, Savinova OV, Nikolaeva SN, Boreko EI, Flekhter OB (2003). Antiviral activity of betulin, betulinic and betulonic acids against some enveloped and non-enveloped viruses. Fitoterapia.

[9] Shamsabadipour S, Ghanadian M, Saeedi H, Rahimnejad MR, Mohammadi-Kamalabadi M, Ayatollahi SM, Salimzadeh L (2013). Triterpenes and steroids from *Euphorbia denticulata* Lam. with anti-herpes symplex virus activity. Iran J Pharm Res.

[10] Tene M, Ndontsa BL, Tane P, Tamokou JF, Kuiate JR (2009). Antimicrobial diterpenoids and triterpenoids from the stem bark of *Croton macrostachys*. Int J Biol Chem Sci.

[11] Hata K, Hori K, Ogasawara H, Takahashi S (2003). Anti-leukemia activities of Lup-28-al-20(29)-en-3-one, a lupane triterpene. Toxicol Lett.

[12] Gauthier C, Legault J, Lebrun M, Dufour P, Pichette A (2006). Glycosidation of lupane-type triterpenoids as potent in vitro cytotoxic agents. Bioorg Med Chem.

[13] Liu M, Yang S, Jin L, Hu D, Wu Z, Yang S (2012). Chemical constituents of the ethyl acetate extract of *Belamcanda chinensis* (L.) DC roots and their antitumour activities. Molecules.

[14] Yang S, Liu M, Liang N, Zhao Q, Zhang Y, Xue W, Yang S (2013). Discovery and antitumor activities of constituents from *Cyrtomium fortumei* (J.) Smith rhizomes. Chem Cent J.

[15] Zuco V, Supino R, Righetti SC, Cleris L, Marchesi E, Gambacorti-Passerini C, Formelli F (2002). Selective cytotoxicity of betulinic acid on tumor cell lines, but not on normal cells. Cancer Lett.

[16] Boryczka S, Bębenek E, Wietrzyk J, Kempińska K, Jastrzębska M, Kusz J, Nowak M (2013). Synthesis, structure and cytotoxic activity of new acetylenic derivatives of betulin. Molecules.

[17] Rzeski W, Stepulak A, Szymański M, Juszczak M, Grabarska A, Sifringer M, Kaczor J, Kandefer-Szerszeń M (2009). Betulin elicits anti-cancer effects in tumour primary cultures and cell lines *in vitro*. Basic Clin Pharmacol Toxicol.

[18] ICCVAM (Interagency Coordinating Committee on the Validation of Alternative Methods). Guidance document on using *in vitro* data to estimate *in vivo* starting doses for acute toxicity. Research Triangle Park: NIEHS. 2001; https://ntp.niehs.nih.gov/iccvam/docs/acutetox_docs/guidance0801/iv_guide.pdf.

[19] Vinken M, Blaauboer BJ (2017). In vitro testing of basal cytotoxicity: establishment of an adverse outcome pathway from chemical insult to cell death. Toxicol In Vitro.

[20] Castaño A, Gómez-Lechón MJ (2005). Comparison of basal cytotoxicity data between mammalian and fish cell lines: a literature survey. Toxicol In Vitro.

[21] Poornavaishnavi C, Gowthami R, Srikanth K, Bramhachari PV, Venkatramaiah N (2019). Nickel nanoparticles induces cytotoxicity, cell morphology and oxidative stress in bluegill sunfish (BF-2) cells. Appl Surf Sci.

[22] Srikanth K, Sundar LS, Pereira E, Duarte AC (2018). Graphene oxide induces cytotoxicity and oxidative stress in bluegill sunfish cells. J Appl Toxicol.

[23] Adler S, Pellizzer C, Paparella M, Hartung T, Bremer S (2006). The effects of solvents on embryonic stem cell differentiation. Toxicol In Vitro.

[24] Singh M, McKenzie K, Ma X (2017). Effect of dimethyl sulfoxide on in vitro proliferation of skin fibroblast cells. J Biotech Res.

[25] Jamalzadeh L, Ghafoori H, Sariri R, Rabuti H, Nasirzade J, Hasani H, Aghamaali MR (2016). Cytotoxic effect of some common organic solvents on MCF-7, Raw-264.7 and human umbilical vein endothelial cells. Avicenna J Med Biochem.

[26] Vichai V, Kirtikara K (2006). Sulforhodamine B colorimetric assay for cytotoxicity screening. Nat Protoc.

[27] Pfarr K, Danciu C, Arlt O, Neske C, Dehelean C, Pfeilschifter JM, Radeke HH (2015). Simultaneous and dose dependent melanoma cytotoxic and immune stimulatory activity of betulin. PloS One.

[28] Lin WY, Sadhasivam S, Lin FH (2009). The dose dependent effects of betulin on porcine chondrocytes. Process Biochem.

[29] Mosmann T. Rapid colorimetric assay for cellular growth and survival: application to proliferation and cytotoxicity assays. J Immunol Methods. 1983;65:55-63. 10.1016/0022-1759(83)90303-4 6606682

[30] Małaczewska J, Siwicki AK. Commercial metal-based nanocolloids – evaluation of cytotoxicity. Bull Vet Inst Pulawy. 2015;59:115-122.

[31] Borenfreund E, Puerner JA. A simple quantitative procedure using monolayer culture for toxicity assays. J Tissue Cult Meth. 1984;9:7-9.

[32] Decker T, Lohmann-Matthes M. A quick and simple method for the quantitation of lactate dehydrogenase release in measurements of cellular cytotoxicity and tumor necrosis factor (TNF) activity. J Immunol Methods. 1988;115:61-69. 10.1016/0022-1759(88)90310-9 3192948

[33] Skehan P, Storens R, Scudiero D, Monks A, McMahon J, Warren JT, Bokesch H, Kenney S, Boyd MR. New colorimetric cytotoxicity assay for anticancer-drug screening. J Natl Cancer Inst. 1990;82:1107-1112. 10.1093/jnci/82.13.1107 2359136

[34] Quest Graph™ ED50 Calculator. AAT Bioquest, Inc, 09 Feb. 2021, https://www.aatbio.com/tools/ed50-calculator.

