# Supplementary figures and images for: High cytotoxicity of betulin towards fish and murine fibroblasts: Is betulin safe for nonneoplastic cells?
Source: BMC Vet Res. 2021 May 25;17:198. doi: 10.1186/s12917-021-02905-x (PMC8152350; doi:10.1186/s12917-021-02905-x)

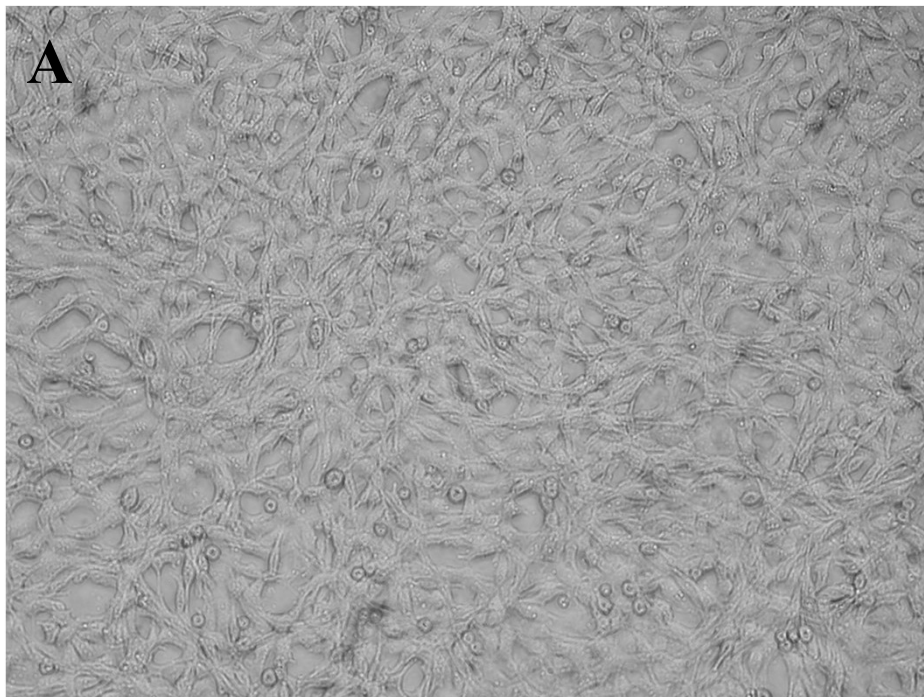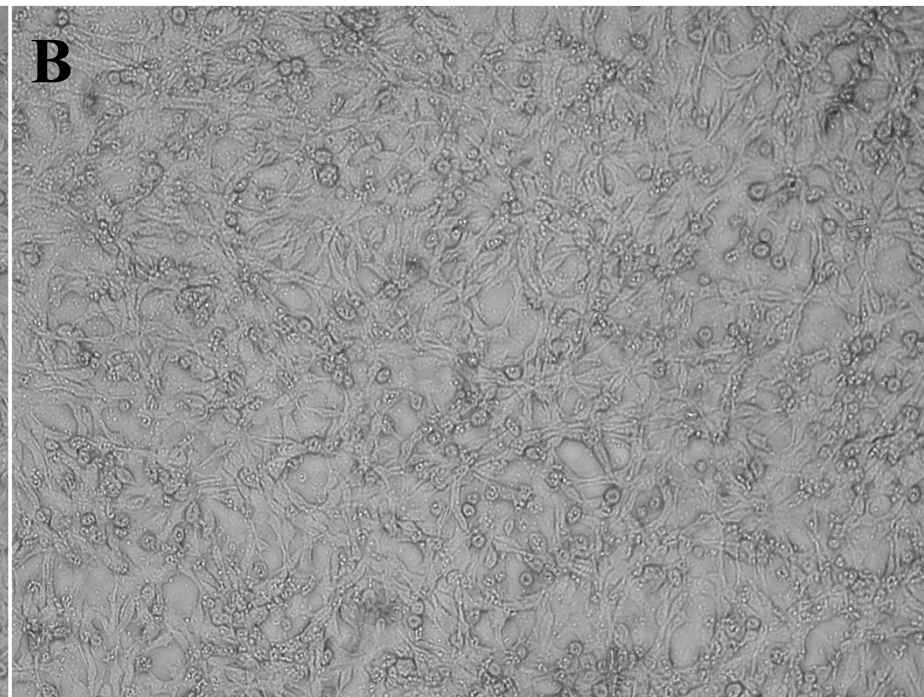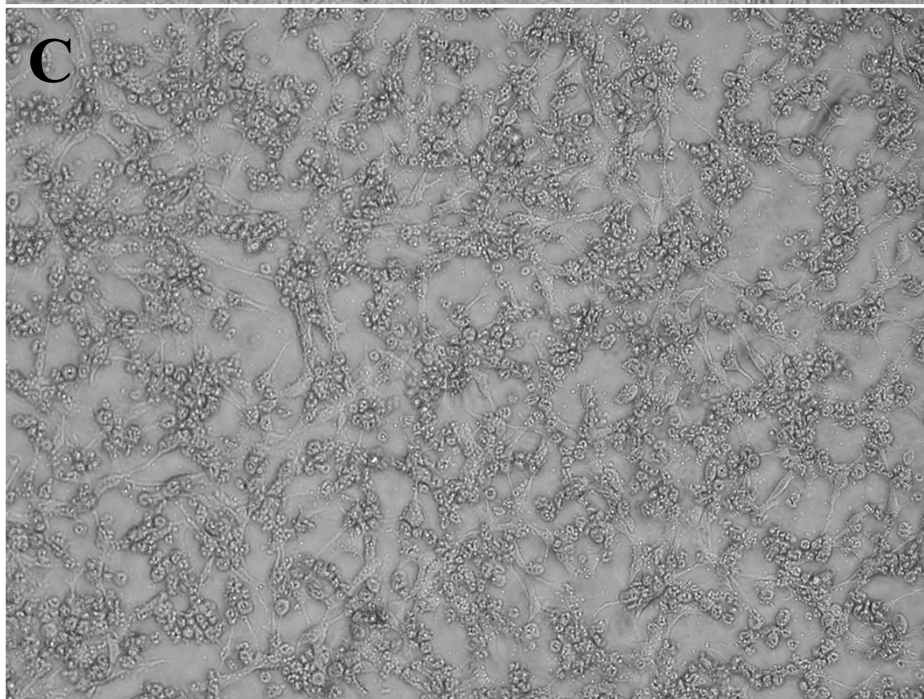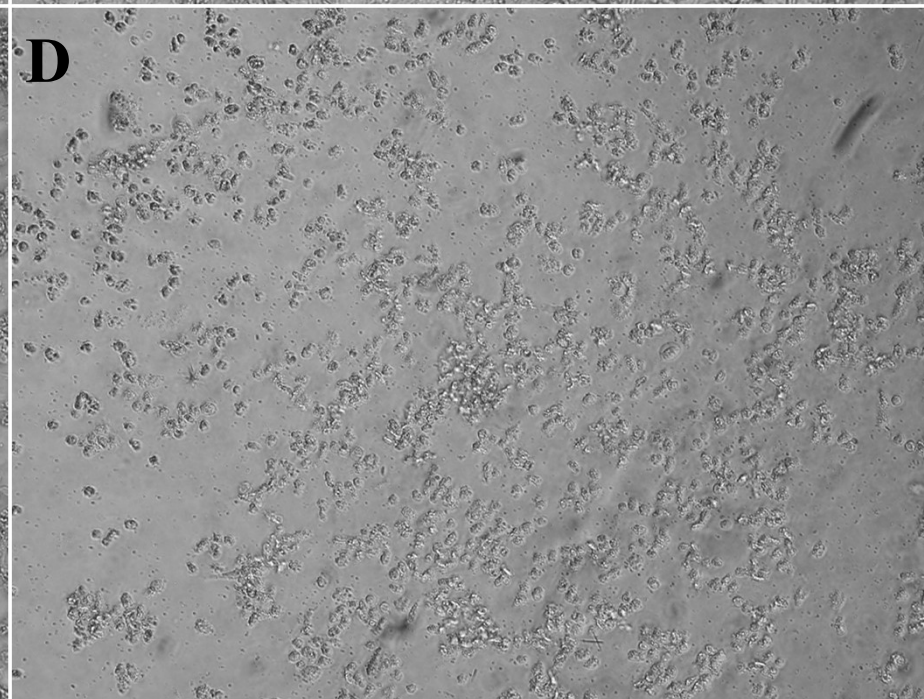

Supplement: Supplementary file 1 — Additional file 1. Representative morphology of BF-2 cells treated for 72 h with different concentrations of betulin (A) control (untreated) (B) betulin 0.976 µg/mL (C) betulin 3.9 µg/mL (D) betulin 15.625 µg/mL (magnification, x100) (PDF). [file 12917_2021_2905_MOESM1_ESM.pdf]

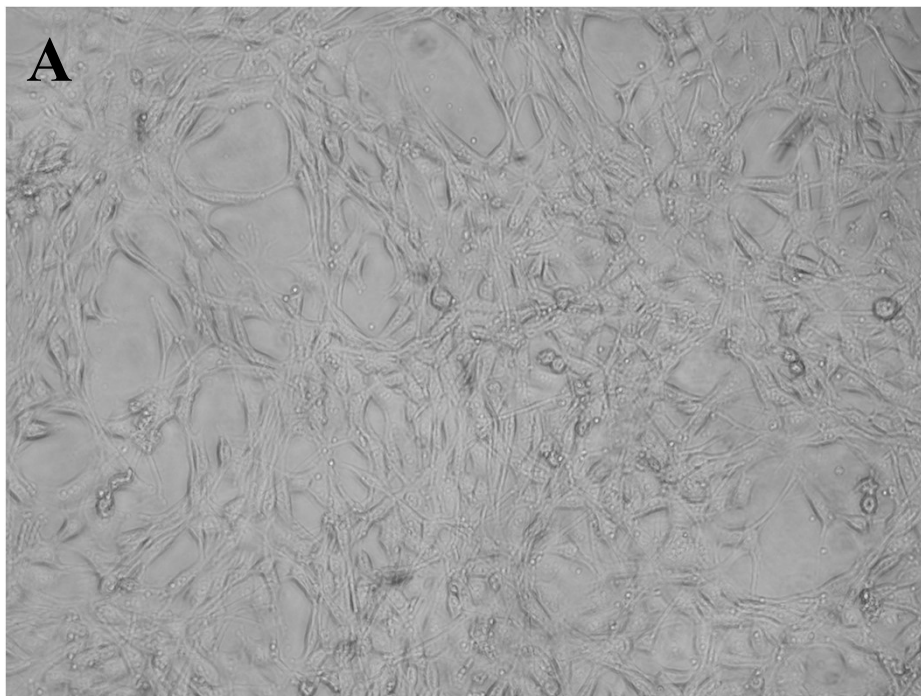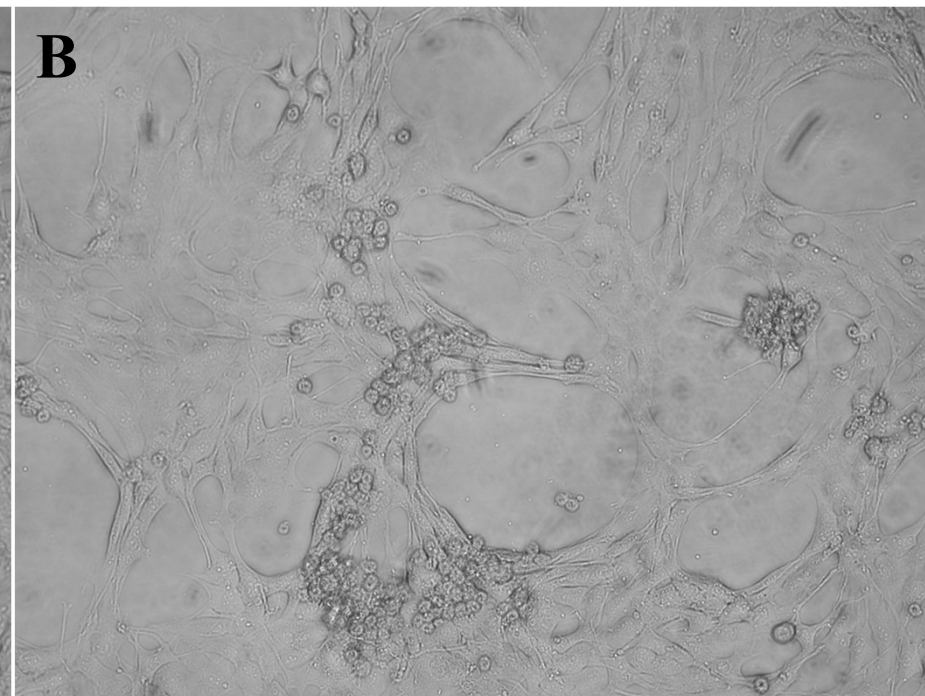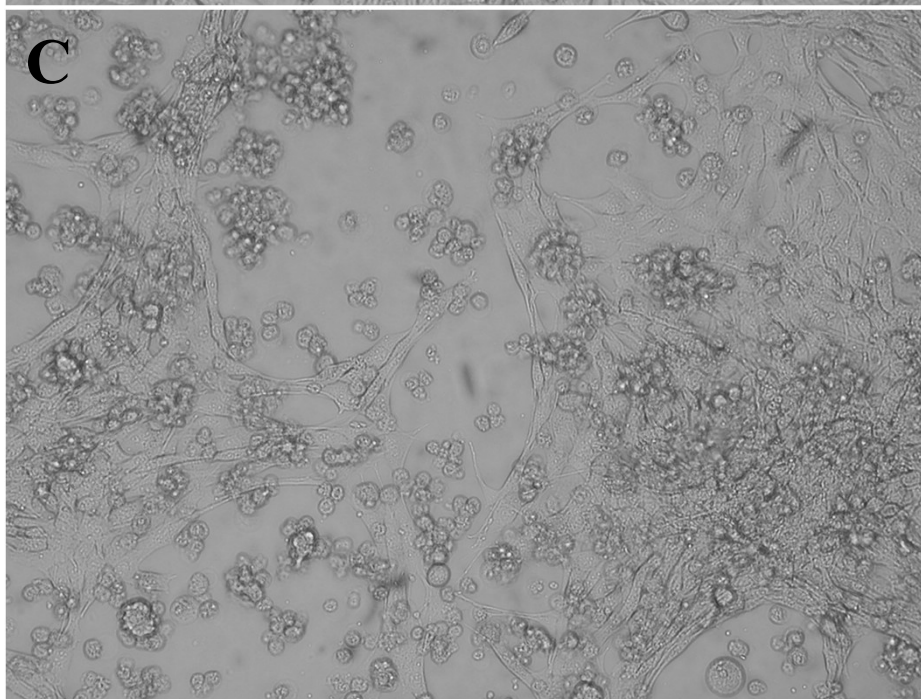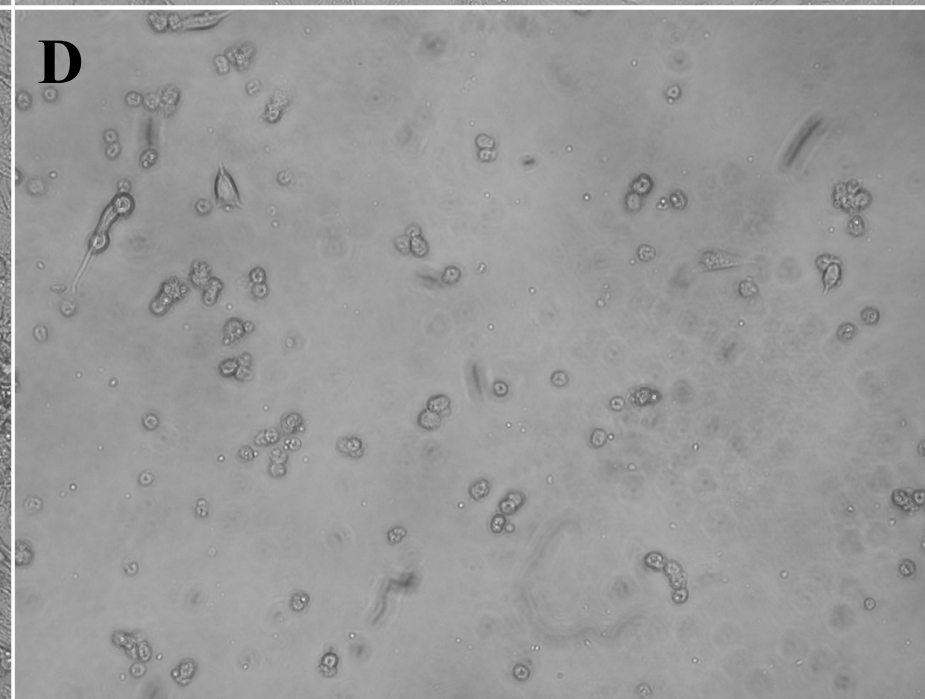

Supplement: Supplementary file 2 — Additional file 2. Representative morphology of NIH 3T3 cells treated for 72 h with different concentrations of betulin (A) control (untreated) (B) betulin 0.976 µg/mL (C) betulin 3.9 µg/mL (D) betulin 15.625 µg/mL (magnification, x100) (PDF). [file 12917_2021_2905_MOESM2_ESM.pdf]
